# Construction of a new membrane bed biofilm reactor and yttria-stabilized zirconia for removing heavy metal pollutants†

**DOI:** 10.1039/d3ra08262h

**Published:** 2024-03-08

**Authors:** Maryam Jahandust, Akbar Esmaeili

**Affiliations:** a Department of Chemical Engineering, North Tehran Branch, Islamic Azad University P.O. Box 1651153311 Tehran Iran akbaresmaeili@yahoo.com +98-21-77009848 +98-912-148-4813

## Abstract

The objective is to design a reactor with a composite new membrane bed biofilm reactor and yttria-stabilized zirconia. We constructed a valuable reactor using response surface methodology (RSM) for process optimization. The present system can remove heavy metal Pb from wastewater using a two-part biofilm reactor: the first reactor, which includes active sludge and media, was investigated; then, the second part, which includes membranes, was made. The amount of heavy metal removed from the effluent was measured at different pH and contact time. The results obtained from this study showed that the optimum conditions for obtaining the optimal removal efficiency separately, with a lead value of 40 mg L^−1^ for the MBBR reactor, had the highest removal value of 55% and for the membrane with an input lead value of 20 ppm at pH = 12 call time 30 minutes equal to 85%. All analyses in this article have been repeated numerous times to prove the repeatability.

## Abstract 2

We construct a membrane bed biofilm reactor combined with yttria-stabilized zirconia to remove heavy metal Pb from wastewater.

## Graphical abstract

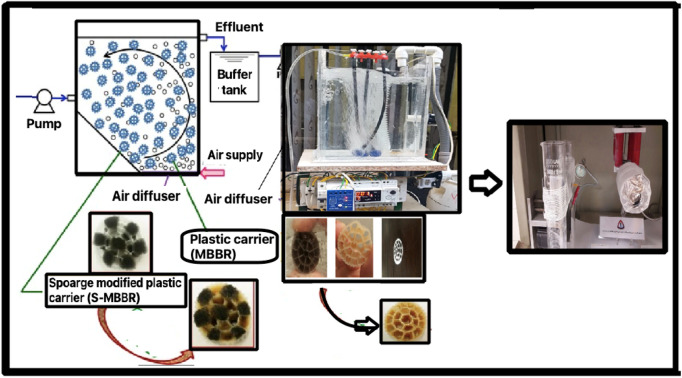

## 1. Introduction

Industrial and technological production of heavy metals has become a fundamental and serious problem for humans and the environment.^1–3^ With increasing development of industries that cause toxic heavy metal pollution, solving this is a fundamental problem for society.^4^ The conventional treatment methods to remove heavy metals are biological, chemical precipitation, filtration, flocculation, membrane filtrations, ion exchange, and electrolysis.^5–7^ Most research shows that natural treatment is an economic and environmentally-friendly method.^8^ Metals are known toxicants, and exposure can damage multiple organs. One such toxic heavy metal is Pb. Based on experimental evidence, children exposed to Pb have altered brain volumes.^5,9–11^ In recent years, biofilm technology has proved its ability to remove wastewater pollution, and the membrane bed biofilm reactor (MBBR) is a cost-effective technology and a promising solution to this problem.

MBBR is an aerobic system containing mixed and suspended small plastic carriers that a biofilm can grow on.^12,13^ Previous studies^14,15^ reduced Pb mobility in soil with refined bauxite residues (MBRR). Experiments were performed on four Pb-contaminated areas polluted with Pb-based paint waste from different sources. The results show that modification of Pb-contaminated soil by 2 to 20% (w/w) reduces Pb mobility. Removal of heavy metals using these methods needs to consider several factors, such as the concentration of sludge, type of metal(s), their concentration, solubility, wastewater pollution, and pH.^16^ MBBR by membrane biofilm can interfere with biofilm growth; biomass limitations can be overcome.^17^ It makes a sludge more efficient for treating highly polluted wastewater fields^18^ without recycling. In addition, the use of a MBBR biofilm reactor helps improve wastewater treatment. Previously, our concern was membrane reactor design and construction so we could use this process in an industrial pilot. In membrane reactors, the accumulation of solid particles creates a dense layer that reduces the membrane permeability.^19,20^ So, membrane fouling is one of the obstacles to membrane reactor development.^21,22^ The dynamics membrane (DM) application has been a new approach for membrane reactors since 1990.^23^ In the DM process, a cake layer comprises suspended wastewater on a support membrane such as fabric, filter, stainless steel mesh, or non-woven fibers.^24^ The activated sludge method and membrane filtration have many advantages. These include running the system with a mixed solution of suspended solids, good disinfection ability, higher volumetric loading, high effluent quality, and a small footprint.^8^ Many synthetic methods have already been indicated for producing a hollow fiber: chemical vapor deposition, hydrothermal reduction method, template synthesis method, and electrospinning.^25^ A previous paper reports the use of hollow fiber prepared yttrium-stabilized zirconia by treating electrospun precursor fibers at 600 °C for 12 h.^26^ Here, we explore an efficient hybrid of an MBBR and hollow fiber to remove Pb from wastewater. The removal behavior was assessed, and optimal conditions found for the biosorption reaction, essential for extracting heavy metals. Biosorbents such as bacteria, fungi, algae, chitin, zeolite, clay, wood, and coal have been successfully used to remove heavy metals, dyes, and organic compounds from water and wastewater and have several advantages.^2^

Biofilm formation is formed by intercellular (interbacterial) interactions called quorum systems. When the bacterial density in an environment reaches a specific limit, the concentration of these transporter molecules reaches a threshold and induces significant changes in the gene expression level. These changes in the gene expression level affect (induce or suppress) various invasive factors, including the bacteria biofilm—changes in the environment around the microorganism cause the planktonic form to become part of a biofilm. In passing from the planktonic phase to the biofilm phase, the gene expression in the bacterial cell undergoes many changes.^27^ The bacterium is enclosed in a self-made extracellular matrix in the biofilm phase. This matrix constitutes 30% of the volume of the biofilm mass. The task of this extracellular matrix is to maintain a three-dimensional structure of the biofilm. The heavy metal trapped inside the matrix provides the nutritional needs of the bacteria in the biofilm.^28^ Wastewater is also provided to maintain the bacteria due to the polysaccharide matrix's hydrophilic properties. The present study uses a biofilm reactor to remove two heavy metals from effluent.

## 2. Materials and methods

### 2.1. Materials

ZrOCl_2_·8H_2_O (Merck, Germany), Y(NO_3_)_2_ (Sigma-Aldrich, USA), polyvinyl pyrrolidine (PVP) (Merck, Germany), and C_2_H_5_OH (Merck, Germany) were purchased from TEB-AZUMA in Canada; used for the synthesis of 8YSZ and synthetic wastewater.

### 2.2. Preparation of 8YSZ membranes

**Fig. 1:**
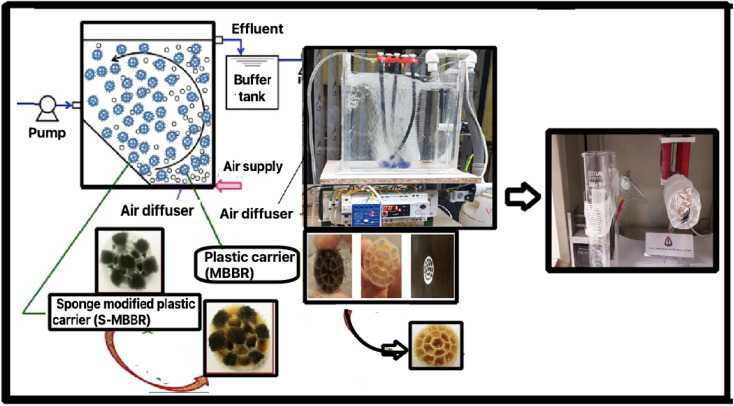
The process of synthesizing the reactor with a new membrane for the MBBR and yttria-stabilized zirconia (8-YSZ).

Based on the sol–gel process, ZrOCl_2_ was dissolved in 20 mL of deionized water (solution number 1). Then, to make yttrium(iii) nitrate, 0.8 g was dissolved in nitric acid and placed under the laboratory hood on a heater for 4 hours to evaporate the acid to slowly obtain crystalline solids (solution number 2). Next, the PVP polymer was dissolved in C_2_H_5_OH and stirred using a magnetic stirrer for 30 minutes (solution no. 3). Finally, we added solution number 3 to solution 2, and placed the resulting mixture on a magnetic stirrer for 4 hours at room temperature^26^ (Fig. 1).

The plan and flow diagram of the pilot used are presented in Fig. 1. In this research, active west media was used to conduct experiments related to the membrane. The inlet is connected to a centrifugal electro-pump device to feed the reactor from a tank with a volume of 50 liters. In the pilot, an electrode-level control system is used. When the fluid level in the reactor goes down, the pumping command is given to the pump, and when the fluid level rises, it provides a shutdown command to the raw sewage feed pump. The dimensions of the suspended growth bioreactor are length = 35 cm, width = 26 cm, total reactor volume ≈ 24 liters, fluid volume ≈ 19 liters. The MBBR pilot aeration section uses floating media made of polypropylene with a specific surface area of 500 square meters per cubic meter. An aeration pump device is utilized, which is connected to the aeration stones on the bottom of the reactor by a hose. A pump similar to the one used at the inlet is used to pump the effluent from the bioreactor.

The solid-state and permeation reaction continued to heat the material for pyrolysis, moisture removal, and intermediates formation. The resulting membrane was placed in the oven for 6 hours and at 600 °C for 12 hours for calcination. Examining the properties of nanoparticles is essential for synthesis. Stability and resistance to hydraulic load, 8YSZ membranes are suitable – hydraulic gear collected in MBBR. The properties of nanoparticles were measured in this work using SEM (TE-SCAN, Czech), X-ray diffraction (Philips PW3040), FT-IR spectra (C88731 spectrophotometer, PerkinElmer, Germany, 400–4000 cm^−1^), and standard methods,^29^ as well as other strategies for determining their size and surface morphology. The most common nanoparticles are ceramic, metal, polymer or semiconductor. In this work, 1000 mg L^−1^ of a lead solution was used as the sample; the salt used was lead nitrate Pb(NO_3_)_2_, and 1.589 g of the salt was dissolved in distilled water, bringing the volume to 1000 mL.

A solution containing 20 mg L^−1^ of lead, according to a design using Design-Expert® software (see Section 2.8.), was placed on the shaker for various times and pH.1
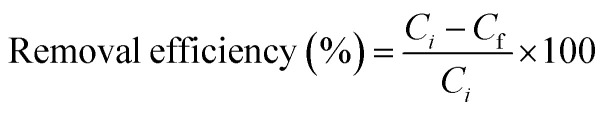
where *C*_i_ and *C*_f_ are the initial and final concentrations (mg L^−1^) of lead, respectively. The effect of different operating parameters on lead removal efficiency, such as pH and contact time, were investigated and optimized.

### 2.3. Pilot specification

**Fig. 2:**
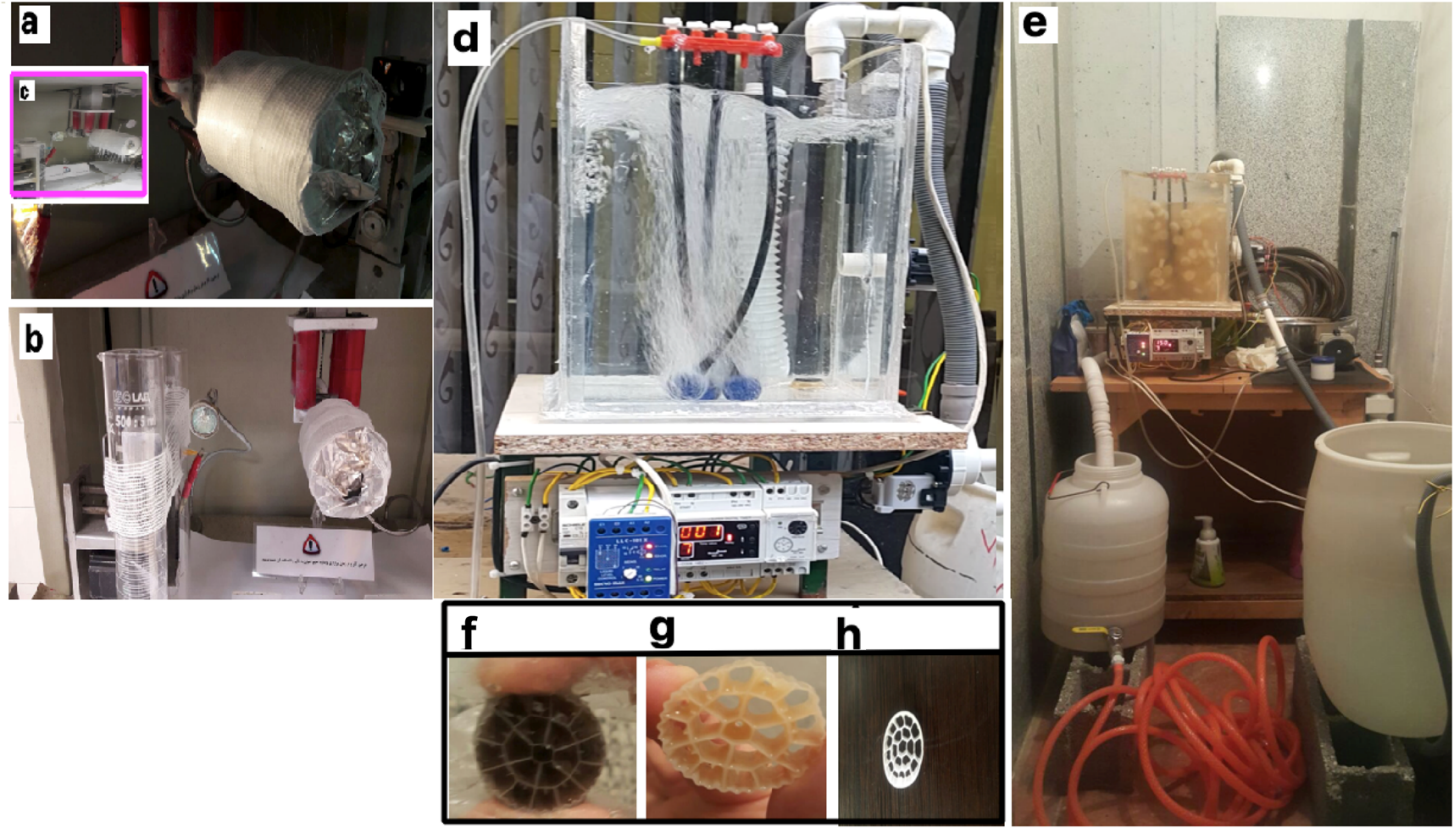
Electrospinning system of 8YSZ: (a) electrospinning machine with bandage, (b) applying nanofibers to the bandage in the electrospinning machine, (c) applying nanofibers to aluminum in the electrospinning machine. (d and e) MBBR and 8YSZ process for removal of lead from wastewater: (d) pilot before launch, (e) pilot after starting work. (f–h) Packing media: (f) before the formation of sludge, (g) three weeks after starting the reactor, (h) two months after starting the reactor.

The actual layout of the lab-scale MBBR is shown in Fig. 2a and b.

Fig. 2a–c show the electrospinning system of 8YSZ: (a) the electrospinning machine with a bandage, (b) the addition of nanofibers to the bandage in the electrospinning machine, (c) the addition of nanofibers to the aluminum in the electrospinning machine. Fig. 2d and e present the MBBR and 8YSZ process for the removal of lead from wastewater: (d) pilot before launch, (e) pilot after starting work. Fig. 2f–h describe the packing media: (f) before the formation of sludge, (g) three weeks after starting the reactor, and (h) two months after starting the reactor.

The length = 35 cm, width = 26 cm, total reactor volume ≈ 24 L, fluid volume ≈ 19 L, has a capacity of 24 liters and a working volume of 19 L. There was a bulk density of 150 kg m^−3^, three air stones (length = 20 cm), and an air compressor. A centrifugal pump (LOWARA-Italy) introduced the wastewater and discharged it *via* a solenoid valve (Uni-D-Taiwan). This study used a pilot containing activated media and human-intervention to perform the membrane tests. The inlet is connected to a centrifugal electric pump to feed the reactor from a tank with a volume of 50 liters. In the pilot, an electrode level control system is used, which gives a shutdown command as the fluid level in the reactor decreases to the raw sewage feed pump, so that the pump starts and the liquid level rises. The dimensions of the suspended growth bioreactor are length = 35 cm, width = 26 cm, total reactor volume ≈ 24 liters, fluid volume ≈19 liters. In the aeration section of the MBBR pilot, floating media with a unique surface of 500 square meters per cubic meter of polypropylene has been used (Fig. 2e). A pilot aeration pump device is used, which is connected to the aeration rocks in the reactor floor by a hose. A pump similar to the one used at the inlet is used to suck the effluent from the bioreactor. Fig. 2f–h show the packing media before the formation of sludge, after three weeks, and after two months, respectively.

### 2.4. Set-up of the pilot process

The first 2/3 volume of the pilot was filled with activated sludge and 1/3 by synthetic wastewater. When the control level activates the start command, the input pump pumps sewage in, then after the fluid reaches the desired level, the input pump receives a shutdown command, and the reactor begins operating. The system was run in batch mode for five days. In this mode, the system worked for eight hours and then stopped for one hour to settle the sludge. The pH of the wastewater was 7–8, optimal for the growth of bacteria at room temperature.

After five days, the reactor was operated continuously in acclimation mode for 45 days.

### 2.5. Adaptation mode

**Fig. 3:**
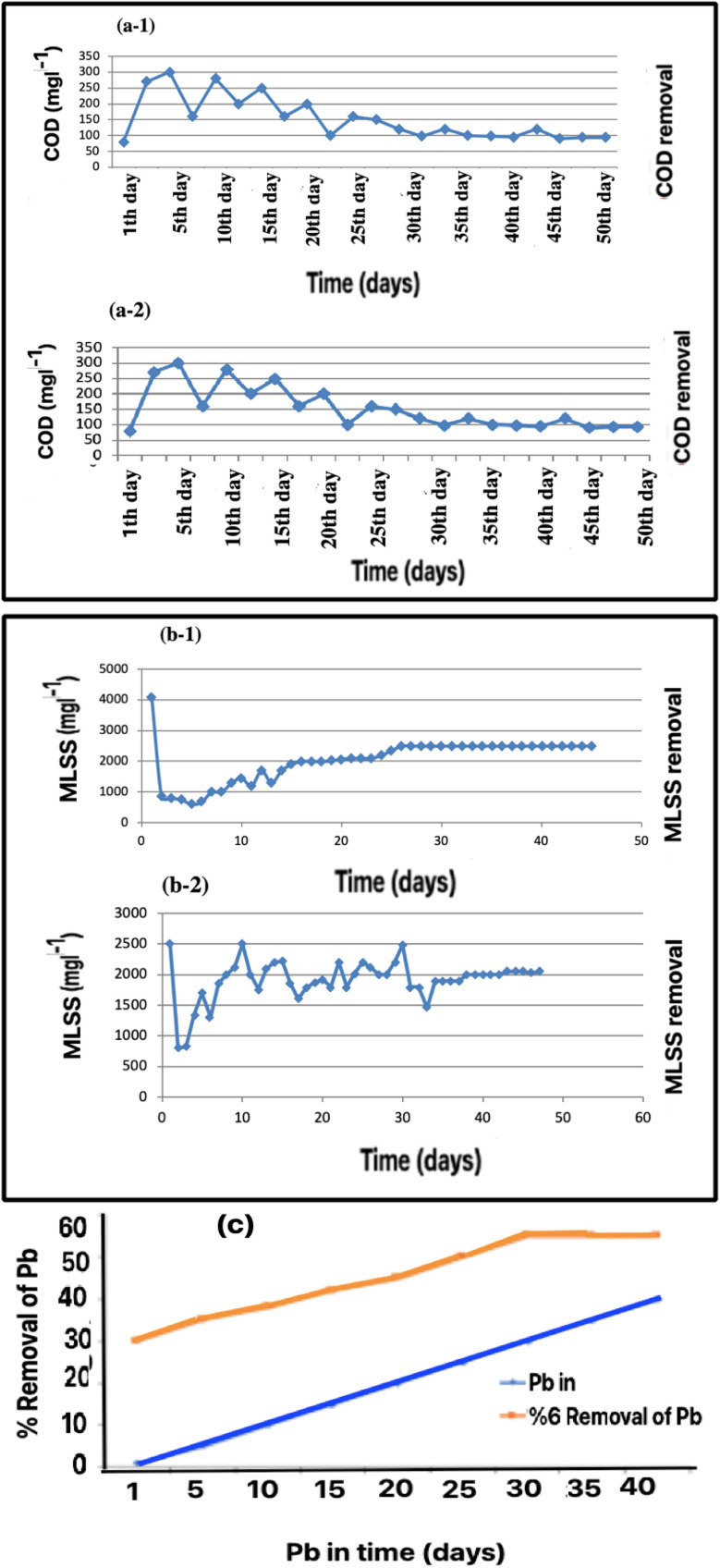
Input, output, and removal: COD: (a-1) during the acclimatization period, (a-2) after Pb addition. MLSS: (b-1) during the acclimatization period, (b-2) after Pb addition. (c) Pb changes in adaptation mode (mg L^−1^).

Adapting microorganisms to environmental conditions is the most critical step in the activated sludge process. Based on our experiments, the system obtained – measured from the suspended solid content – an upward trend in the growth of microorganisms. Mixed-liquid suspended solids (MLSS) show when the development of a microbial structure has increased at the beginning of the process using the aeration and feed measurements. As shown in Fig. 3a-1, COD removal decreased during the first-day, the compatibility period. However, COD removal increased after several days as the biofilm established in the media. In these conditions, the growth of the microorganisms is stable, therefore, the acclimatization is complete and treatment can be started.

### 2.6. Performance review of COD and MLSS after adding Pb

As shown in Fig. 3a-2, after adding Pb in 1 mg of MLSS, the COD decreased, but after a few days, increased. This method uses flotation materials such as iron hydroxide III and aluminum hydroxide III to trap and eventually remove heavy metals such as Pb. It creates a bond between the float and the corresponding ion. Huang *et al.*^30^ used this method to remove heavy metals such as Zn, Cd, Cr, and Pb. They studied the effects of pH and flotation concentration; the appropriate pH for 70% removal efficiency was 9. Also, the best buoyancy concentration of iron hydroxide for removal of 50 mg L^−1^ Pb up to 70% efficiency is equal to 100 mg L^−1^. Sodium dodecyl sulphate (SDS) is also required to remove Pb equivalent to 60 mg L^−1^.

### 2.7. Wastewater characteristics

The activated sludge from a wastewater treatment plant was used to launch the pilot. This sludge was in good condition and contained large sludge flocs with good settling capability. The suspended solids concentration was 4100 mg L^−1^, and the ratio of filamentous microorganisms to floc formations was appropriate. Initially, two-thirds of the reactor volume is filled with sludge and one-third with synthetic wastewater. By placing the liquid level control electrode on the inlet sewage pump, the pump starts, then after the fluid reaches the desired level, the inlet pump turns off the control system. Table 1 shows the quality characteristics of the synthetic wastewater, and Table 2 shows the wastewater feed in the adaptation periods and after the addition of Pb. For Table 1, most of the parameters considered in this research are based on the Standard Methods of Water and Wastewater Testing; a summary of the test method is provided below (APHA, 1999).^31^

**Table 1:** Qualitative characteristics of synthetic wastewater in adaptation periods and after adding Pb

No.	Qualitative characteristics of synthetic wastewater	Amount (mg L^−1^)
1	Chemical oxygen requirements during the adaptation period	250
2	MLSS in the compatibility period	2500
3	Chemical oxygen requirements during operation	500
4	MLSS at runtime	2900

**Table 2:** Synthetic wastewater input ingredients

Description	Milk powder (g)	Glucose (g)	Urea (g)	H_2_PO_4_ (g)
Input synthetic effluent in the adaptation period	3	8.5	0.5	0.2
Synthetic effluent input during driving	6.0	17	1.0	0.4

### 2.8. Optimization of the process

For the experimental design (CCD) we used Design-Expert® software version 12 (Stat-Ease Inc. Minneapolis, USA), to optimize the process.

## 3. Results and discussion

According to our membrane bioreactor process results, it is a suitable method for removing heavy metals from industrial wastewater. Water and wastewater treatment using bioreactors and nanotechnology can lead to a significant improvement in terms of spending money and increasing the efficiency of removing heavy metals. The response surface method was used to optimize the process and evaluate effective parameters such as pH and contact time on lead removal efficiency. The optimal conditions for obtaining lead removal efficiency are a pH equal to 11 and a contact time of 30 minutes. The obtained results prove that the MBBR reactor and self-made membrane hybrid are effective and could provide an important step towards improving the environment.

The MBBR process used in this process has efficiencies that other processes do not have: increasing capacity by converting an activated sludge system to a moving bed reactor and expanding the capacity of an activated sludge system by adding a moving bed biofilm reactor in front of the process – a combination of MBBR as a filter system with activated sludge as a system with suspended solids.^2–4^ One of the methods of removing heavy metals, including lead, from water and wastewater is using chemicals for coagulation and flocculation. These chemicals become heavy after forming a bond with the corresponding metal and settle. Sari and colleagues have studied different coagulants to remove lead from industrial wastewater. They investigated various parameters, such as adsorbent concentration, pH, lead concentration.^32,33^ According to their results, lead absorption increases with increasing pH. The best pH, achieving 98% efficiency, is pH 8. Also, it has been previously shown that the absorption efficiency increases with the increase of adsorbent concentration and lead concentration so that with the rise of adsorbent concentration from 2 g L^−1^ to 40 g L^−1^, the absorption efficiency increases from 48% to 95%.^2,34^

YSZ is a versatile material with a wide range of applications. Compared to conventional powders, YSZ powders have tiny crystal dimensions, superior phase homogeneity, and low-temperature sinter ability.^35^ High pressure can also be used to stabilize YSZ because zirconia under the conditions of high temperature and pressure, transforms into an orthorhombic state, for example, an orthorhombic phase observed in thin foils of yttria-doped zirconia powders.^36^

### 3.1. Membrane bed biofilm reactor (MBBR)

#### 3.1.1. Adaptation period

One of the most critical stages of activated sludge processes is the adaptation period of microorganisms to the environmental conditions. As seen in Fig. 3a and b, after adding activated sludge and artificial wastewater, the reactor faced a decrease in MLSS and an increase in COD at the beginning of the period. However, considering the availability of growth conditions and the suitable aeration, the biofilm layer eventually formed.

After several days, the MLSS and COD reduction reached stable and acceptable conditions. The results obtained from measuring the amount of suspended solid particles and MLSS, which indicate the growth of the microbial structure inside the bioreactor, are shown in Fig. 3a and b. The upward trend related to the development of microorganisms is visible.

As shown in Fig. 3b, MLSS increased in general. Its growth rate increased at the beginning of the aeration and feeding process, and as it progressed, its growth rate stabilized and finally reached a constant value of MLSS. In these conditions, the growth of sludge and microbes reached such a level that the purification operations could be started. In this research, two parts of the pilot system, along with the working method and membrane construction, were investigated in detail. Two MBBR reactors, one for sampling and the other as a control, were used to remove the heavy metals.^2,5^

In the suspended growth activated sludge system, the disposal of sludge and increasing MLSS make it possible to deal with higher organic and hydraulic loads than usual. If necessary, the retention time can be increased by reducing the amount of excess sludge disposed. The conditions for some bacteria, such as nitrate-forming bacteria, were optimized. However, in biofilm systems, there are not many tools to control this process, and it is impossible to control the amount of biological mass in the system accurately. At the same time, the possibility of influencing the types of active species in the system is also slight because there is no practical method to separate part of the formed microbial film from the system.^37^

By increasing the ratio of COD, the COD removal rises to the optimal balance of 0.6. Optimum COD removal efficiency, which reached a COD concentration of 480 mg L^−1^, reduced the amount of lead due to the inhibitory effects. As the hydraulic retention time decreased from 24 to 8 h, the COD removal efficiency gradually reduced due to the increased hydraulic loading rate. The MBBR system was highly stable against toxic and hydraulic shock. The more chemicals there are in the wastewater, the more oxygen is needed for their oxidation, which ultimately increases the value of the COD parameter in the wastewater. In other words, the higher the chemical oxygen demand of wastewater, the more oxidizing chemicals it contains and the more polluted it is.^2^

#### 3.1.2. MLSS and COD changes after lead addition

After the adaptation period when the bioreactor reached a steady state, the system was run for 45 days, adding lead over 5 days. There was an increase in COD with a decrease in lead removal initially. Then with the passage of time and the adaptation of the microorganisms to the environment, an increase in lead removal and a reduction in output COD was found.

These results show that the MLSS concentration was the leading cause of the MBBR characteristics. Some studies have reported that sedimentation is independent of the MLSS concentration when it reached a very high value.^38^ The tested MLSS concentration range in the present study differed from the values in previous literature, the optimal performance was for MLSS at 2500–3500 mg L^−1^. For practical application, evaluation using analytical modeling is suggested.

### 3.2. Optimization using response surface methodology (RSM)

In the studies that have been done samplings and controlled MBBR reactors to remove the heavy metals,^2,5^ the studies investigated two reactors with synthetic wastewater with a total COD of 800 mg L^−1^ at different residence times and a temperature of 21 °C. Increasing the phenolic COD to authentic COD increased the COD removal efficiency to the optimal ratio of 0.6.

This optimal ratio reduces the COD removal efficiency when the phenolic COD concentration reaches 480 mg L^−1^ due to the inhibitory effects of phenol. As the hydraulic retention time decreases from 24 to 8 hours, the COD removal efficiency gradually decreases due to the increased hydraulic loading rate. Examination of microorganisms on the internal and external surface of the biofilm shows a high concentration of filaments. But no filamentous bacteria were found in the mixed liquid and the reactor effluent. Therefore, this reactor's bulking problem was not observed.^2,39^ RSM was used to determine the optimal conditions for removing lead from synthetic wastewater with two variables/factors investigated. Thirteen experiments were designed with the Design-Expert® statistical software (Table 3). The pH 1 and 2 acidic process is unsuitable for the biological system. The optimal conditions for lead removal efficiency are a pH equal to 11 and a contact time of 30 minutes. The results prove the hybrid MBBR reactor and its membrane work in this field (Table 3). The data are based on RSM optimization for the experimental design section.

**Table 3:** The central composite design matrix for the removal of Pb derived from RSM

No.	Std	Run	Factor 1	Factor 2	Response 1
			A: pH	B: contact time	RE (%)
1	11	1	7.00	75.00	54
2	13	2	7.00	75.00	54
3	4	3	12.00	120.00	85
4	12	4	7.00	75.00	54
5	7	5	7.00	11.36	51
6	2	6	12.00	30.00	85
7	8	7	7.00	138.64	75
8	3	8	2.00	120.00	10
9	10	9	7.00	75.00	54
10	1	10	2.00	30.00	25

**Fig. 4:**
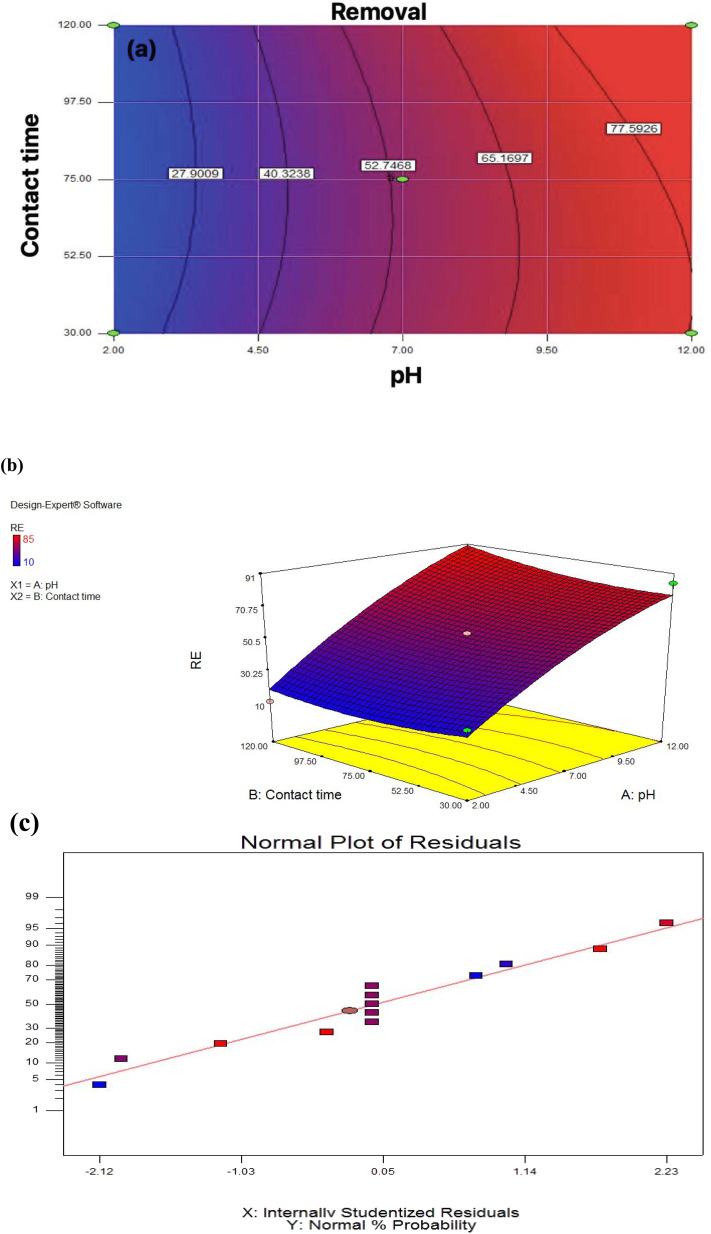
(a) Two-dimensional graph, (b) three-dimensional graphs, (c) standard plot of residual removal efficiency with pH.

To determine the relationship between independent variables and response, two-dimensional and three-dimensional graphs of the response surface were considered. According to Fig. 4a and b, the removal efficiency of the Pb by the membrane would increase with increasing pH and decrease significantly with pH reduction. Also, at pH 7, Pb elimination efficiency increases with increasing contact time. According to this scheme, the optimal removal of Pb at pH 12 and a contact time of 30 minutes, equals 85%. Comparing the current study with previous studies shows that fiber nanoparticles effectively remove Pb from industrial wastewater. A previous study using biomass to remove pollutants from industrial wastewater found pH > 3 was optimal.^40^ The results obtained from the statistical analysis are shown in Table 4. Explanation coefficient *R*^2^ is commonly used to check the accuracy of the ANOVA test. In this section, all calculations related to the analysis of variance with the selected ANOVA test are presented. The significance of the model is also found to be significant.

**Table 4:** ANOVA results of the quadratic model for removal efficiency of Pb.

Source	df	*F* value	*P*-value prob > *F*
Model	5	32.88	0.0001
A: pH	1	159.60	<0.0001
B: Contact time	1	0.96	0.3609

Regression analysis is used to evaluate a linear relationship between test results. A linear relationship is, in general, evaluated over the range of the analytical procedure. The data obtained from analysis of the solutions prepared at a range of different concentration levels is habitually investigated by plotting on a graph. Linear regression evaluates the relationship between two variables by fitting a linear equation to the observed data.

The results obtained from the statistical analysis are shown in Table S1.†*R*^2^ explanation coefficient is commonly used to check the validity of the model. In this section, all calculations related to analysis of variance with the selected model are presented. The significance of the model is also stated. Fig. 4c, to shown the selected model is significant. The response with high confidence coefficients was significant, it indicates that all the models represent the trend of the data well. The closer the *R*^2^ value is to one, the more powerful the fitted model is for describing response changes as a function of independent variables. According to the table for the efficiency factor, the quadratic model was statistically significant, *P* ≤ 0.05; other significant terms of the model included the interaction of pH and contact time. The high value of *R*^2^ (0.9592) and Adjusted *R*^2^ (0.9300) also indicated the high power of the quadratic model for the prediction.

The results show that the size of the membrane was effective in removing the pollutant and this is due to the good cohesion between the particles and the membrane, the presence of lead causes precipitation because the increase in membrane size causes accumulation between particles and the particle size becomes larger.^41^ From the results obtained in Table 3 and 4, it can be seen that the optimal conditions for obtaining optimal lead removal efficiency are pH 12 and a contact time of 30 minutes.

As shown in Fig. 4c, the model chosen is essential. *R*^2^ is corrected to ensure that the model can accurately estimate the numbers; the *R*^2^ explanation coefficient is expressed as the ratio of variations described by the model to total variations, which is a criterion of fit fitness. Fig. 4c shows how to follow the residue from a normal state. Using the statistical method of the response surface, the last equation that shows the experimental relationship between the test variables and the efficiency percentage is encoded.^1,42,43^

2 *Y* = 54.21 + 32.09*X*_1_ + 2.37*X*_2_ +3.75*X*_1_*X*_2_ − 6.52*X*_1_*X*_2_ + 4.12*X*_1_*X*_2_

In this equation, *Y*, is the removal efficiency of Pb, *X*_1_, and *X*_2_ is the pH and contact time, respectively.

According to the biofilm reactor process results, it is a suitable method for removing heavy metals from industrial wastewater. Nanofiber membranes were investigated for removing Pb metal from industrial wastewater. Bioreactors and nanotechnology for water and wastewater treatment can have significant advances in terms of cost and increase the removal efficiency of heavy metals. The surface response method was used to optimize the process and evaluate the effective parameters such as pH, contact time, and Pb elimination efficiency. The optimum conditions for obtaining the optimal removal rate of Pb are pH 12 and a contact time of 30 minutes. The results confirm that the hybrid MBBR reactor and self-made membrane are practical.

### 3.3. FT-IR analysis

**Fig. 5:**
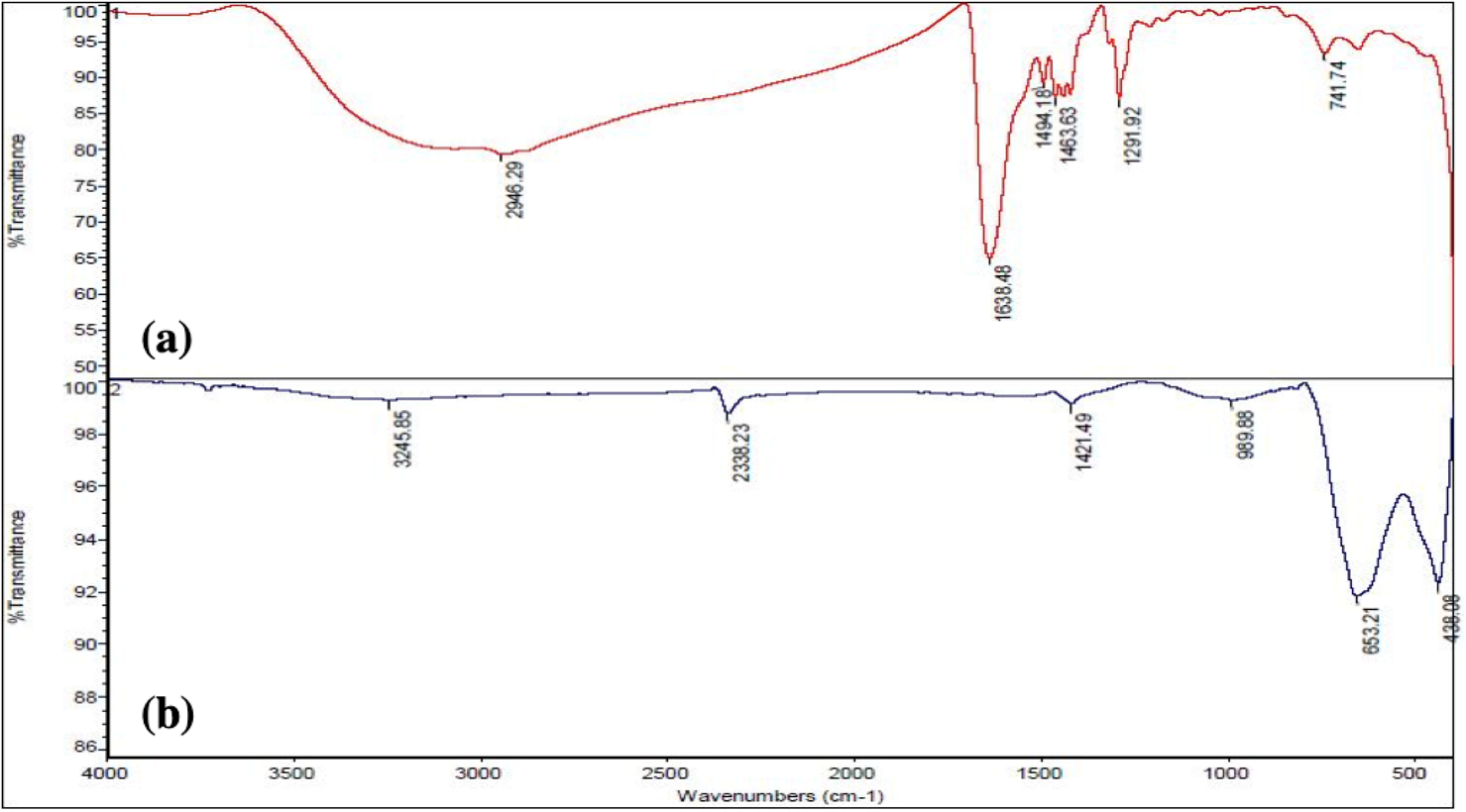
FT-IR spectra of 8YSZ: (a) before and (b) after calcination.

The FT-IR in Fig. 5a shows absorption at 2500–3500 cm^−1^ associated with O–H stretching and intermolecular hydrogen bonding.^44^ Recent articles show similar absorption at 3000–3500 cm^−1^ for O–H stretch. The peak of 1638 cm^−1^ is related to C=O ester groups. The mountain-like peaks at 1494–1463 cm^−1^ are the symmetric and asymmetric stretching of the carboxylate group. In other articles,^10,39^ the carboxylate group presents a similar frequency. The peak at 1291 cm^−1^ is related to the C–OH group, and that at 741 cm^−1^ might be the M–O starching mode.^45^ After calcination of 8YSZ at 600 °C (Fig. 5b), the peak at 3245 cm^−1^ might be due to the Zr–OH asymmetric vibrational frequencies.^46^ Also, the presence of a peak in the wave number below 1000 cm^−1^ is seen, which is related to the vibrational mode of the cation–oxygen bond M–O, M = (Y, Zr).^15^ As shown in Fig. 5, with the increase of the amount of metal in the samples, the intensity of the vibrational peaks of water molecules and hydroxyl groups increases, which could be due to the high water absorption by the metal.

### 3.4. SEM analysis

8YSZ powder is prepared through a chemical route, and weak aggregation occurs. The SEM image shows the calcined 8YSZ gel powder.

**Fig. 6:**
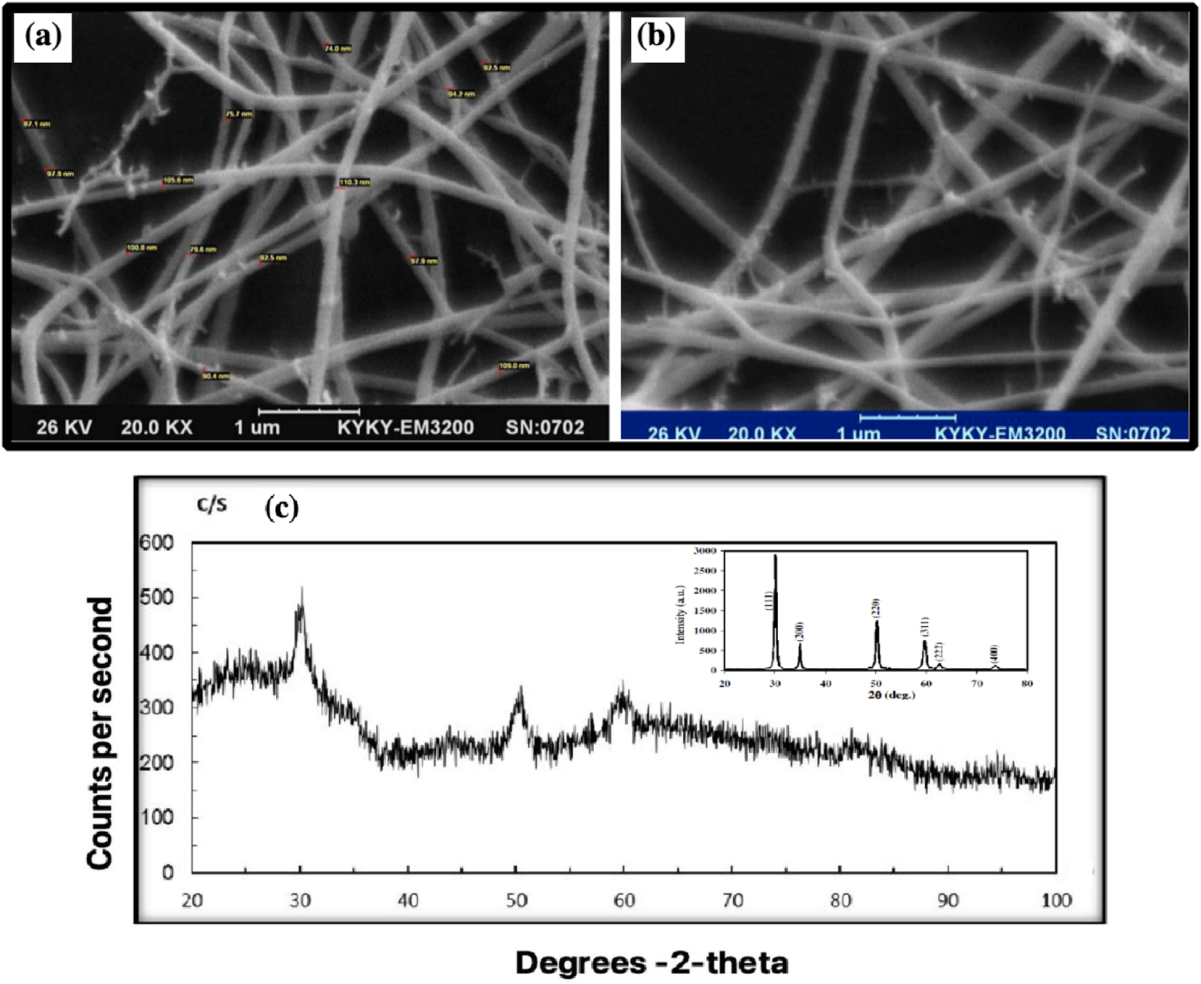
(a and b) SEM pattern of 8YSZ. (c) XRD patterns of the calcined 8YSZ powder.

The ZrO_2_ cubic nanocrystalline powder has a spherical morphology with an average 74–110 nm diameter. The largest cluster is a few micrometers in diameter.^47^ The difference in grain size of 8YSZ may be due to differences in the aggregation of powder particles or grain growth due to the formation of a liquid phase.^48^ SEM was used to determine the morphology and particle size of 8YSZ after calcination at 600 °C for 12 nm.^49^ The nanofiber size was 74–110 nm (Fig. 6a and b). Comparing with previous articles shows the same morphology and size of particles.^50,51^ Both SEM images were taken from the same sample.

### 3.5. X-ray diffraction (XRD) analysis

Fig. 6c shows the XRD structure and purity of 8YSZ after calcination at 600 °C for 8YSZ. It has peaks corresponding to (30), (50), (60), and (80) cubic zirconia. The Scherrer formula can be applied to calculate the average size of crystallite from the prominent peaks at 2*θ* for 30, 50, and 60.^26,52^ The XRD pattern of 8YSZ indicates the core-cube structure, where the six characteristic peaks are consistent with the indicators of previous reports (Fig. 6c).^53,54^ Due to the similarities between the cubic and quadrilateral zirconia field 49 phases, the prepared coatings are composed of steps. The coating grade does not affect the parameters, and all layers show the same form. Our results are consistent with the reported data.^55,56^

The Fig. 6c inset shows the peaks corresponding to the (111), (200), (220) and (311) reflections of cubic zirconia. The particles are seen as clusters and are almost spherical with particle sizes in the range 200 to 50 nm. The calcined powders are still amorphous but the crystallinity of the cubic phase is significantly improved. Fig. 6c also confirms that ranging from 673 to 773 K causes the formation of cubic YSZ nanoparticles.

## 4. Conclusion

The MBBR system’s operational control parameters are relatively simple, all that is required is to monitor the reactor *via* continuous automated control, testing the daily organic COD feed (proxy for BOD), and checking the nutrient levels in the system using a dip strip. Several installation options ensure maximum exposure of MLSS to the media. The bacteria adhere to the media while digesting waste from the plant effluent stream. BOD and COD were investigated in this research. Heavy metal-contaminated wastewater was treated for metal ions reducing the turbidity using a MBBR and 8YSZ process to remove Pb from wastewater. This system reduces energy costs because the fluid flows using gravity and air pressure rather than a stirred reactor. In the present study, heavy Pb metal was removed from wastewater using a two-part biofilm reactor. The COD and heavy metal removal from the effluent were measured under various pH and contact times. The results obtained from this study showed that the optimum conditions for Pb removal: a Pb value of 40 mg L^−1^ for the MBBR reactor, provided the highest removal value of 55%; and for the membrane with an input Pb value of 20 mg L^−1^ at pH 12 and a contact time of 30 minutes provided removal up to 85%.

## Abbreviations

8-YSZ: Yttria-stabilized zirconia
MBBR: Membrane bed biofilm reactor
COD: Chemical oxygen demand
PVP: Polyvinyl pyrrolidine
MLSS: Mixed-liquid suspended solids
SEM: Scanning electron microscopes
XRD: X-ray diffraction
FT-IR: Fourier transfer infrared spectroscopy
RSM: Responsive surface method

## Conflicts of interest

There are no conflicts to declare.

## Supplementary Material

RA-014-D3RA08262H-s001
