# Supplementary material for: Construction of a new membrane bed biofilm reactor and yttria-stabilized zirconia for removing heavy metal pollutants
Source: RSC Adv. 2024 Mar 8;14(12):8150–60. doi: 10.1039/d3ra08262h (PMC10921917; doi:10.1039/d3ra08262h)
Supplement: RA-014-D3RA08262H-s001 [file RA-014-D3RA08262H-s001.pdf]

Electronic Supplementary Material (ESI) for RSC Advances.
This journal is © The Royal Society of Chemistry 2024

Supplementary file

Construction of a new membrane bed biofilm reactor and Yttria-stabilized zirconia for removing heavy metal pollutants

Table 1S

Response 1 RE

ANOVA for Response Surface Quadratic Model

Analysis of variance table [Partial sum of squares - Type III]

Source	Sum of Squares	df	Mean Square	F Value	p-value Prob > F	
Model	7714.72	5	1542.94	32.88	0.0001	significant
A-pH	*7489.79*	*1*	*7489.79*	*159.60*	*< 0.0001*	
B-Contact time	*44.85*	*1*	*44.85*	*0.96*	*0.3609*	
AB	*56.25*	*1*	*56.25*	*1.20*	*0.3098*	
A^2	*239.96*	*1*	*239.96*	*5.11*	*0.0582*	
B^2	*119.10*	*1*	*119.10*	*2.54*	*0.1552*	
Residual	328.51	7	46.93			
Lack of Fit	*328.51*	*3*	*109.50*			
Pure Error	*0.000*	*4*	*0.000*			
Cor Total	8043.23	12				
